# Identification and Determination of 1,3-Thiazinane-4-carboxylic Acid in Human Urine—Chromatographic Studies

**DOI:** 10.3390/ijms23020598

**Published:** 2022-01-06

**Authors:** Justyna Piechocka, Natalia Litwicka, Rafał Głowacki

**Affiliations:** Department of Environmental Chemistry, Faculty of Chemistry, University of Lodz, 163 Pomorska Str., 90-236 Łódź, Poland; natalia.litwicka@edu.uni.lodz.pl

**Keywords:** derivatization, formaldehyde, gas chromatography–mass spectrometry, homocysteine, homocysteine thiolactone, human urine, isobutyl chloroformate, liquid–liquid extraction, 1,3-thiazinane-4-carboxylic acid

## Abstract

It is well established that homocysteine (Hcy) and its thiolactone (HTL) are reactive towards aldehydes in an aqueous environment, forming substituted thiazinane carboxylic acids. This report provides evidence that Hcy/HTL and formaldehyde (FA) adduct, namely 1,3-thiazinane-4-carboxylic acid (TCA) is formed in vivo in humans. In order to provide definitive proof, a gas chromatography–mass spectrometry (GC–MS) based method was elaborated to identify and quantify TCA in human urine. The GC–MS assay involves chemical derivatization with isobutyl chloroformate (IBCF) in the presence of pyridine as a catalyst, followed by an ethyl acetate extraction of the obtained isobutyl derivative of TCA (TCA-IBCF). The validity of the method has been demonstrated based upon United States Food and Drug Administration recommendations. The assay linearity was observed within a 1–50 µmol L^−1^ range for TCA in urine, while the lowest concentration on the calibration curve was recognized as the limit of quantification (LOQ). Importantly, the method was successfully applied to urine samples delivered by apparently healthy volunteers (*n* = 15). The GC–MS assay may provide a new analytical tool for routine clinical analysis of the role of TCA in living systems in the near future.

## 1. Introduction

Population aging is undoubtedly one of the most dominant phenomena of our century. In parallel, civilization diseases, to which neurodegenerative diseases, cardiovascular diseases (CVD) and metabolic disorders belong, among others, are increasing in global prevalence. They seriously threaten developing nations as they are one of the most frequent causes of the morbidity and mortality of humans. Despite sustained efforts to research as many diseases as possible, the way to quickly diagnose, prevent, cure or even slow undesirable changes in the human body, resulting in disease, remains substantially unknown.

Among other things, it has been recognized that sulfur-containing compounds, to which homocysteine (Hcy) and its metabolite Hcy thiolactone (HTL) belong, and formaldehyde (FA) are implicated in a number of civilization diseases [1,2,3,4,5,6,7,8,9,10]. In particular, their excess has been recognized as either an initiator or a marker of serious pathogenic processes, despite the fact that Hcy, HTL and FA are found in living systems as normal products [2,11]. For instance, the rise in plasma/urine Hcy and HTL levels is considered to be a risk predictor for morbidity and mortality in CVD, Alzheimer’s disease (AD) and diabetes mellitus (DM) [1,2,3,4,5]. At the same time, it was found that FA levels were elevated in the urine and blood of aging populations, and that these elevations were more prominent in patients suffering from CVD, AD events and DM [6,7,8,9,10]. Therefore, it seems reasonable to assume that factor(s) contributing to Hcy, HTL and FA depletion; factor(s) capable of depleting the precursors of these compounds; or even factors that affect the whole biochemical pathway might be desirable for living organisms. In particular, the possibility of the conversion of Hcy, HTL and FA into a relatively inert compound in vivo is not without practical consideration.

**Figure 1:**
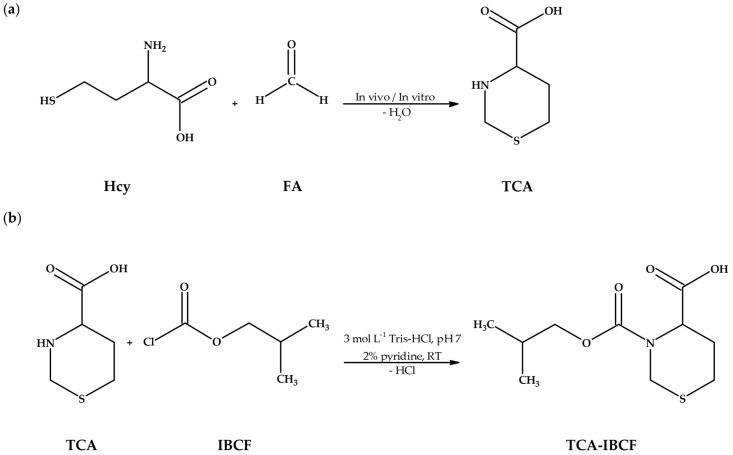
Research project objectives. (**a**) The reaction equation of homocysteine (Hcy) with formaldehyde (FA) providing 1,3-thiazinane-4-carboxylic acid (TCA); (**b**) The chemical derivatization reaction of TCA with isobutyl chloroformate (IBCF) in the presence of pyridine, resulting in formation of an isobutyl derivative of TCA (TCA-IBCF). Abbreviations: FA, formaldehyde; Hcy, homocysteine; IBCF, isobutyl chloroformate; RT, room temperature; TCA, 1,3-thiazinane-4-carboxylic acid; TCA-IBCF, isobutyl derivative of 1,3-thiazinane-4-carboxylic acid; Tris-HCl, tris(hydroxymethyl)aminomethane-hydrochloride acid buffer.

In general, it is well established that amino acids and their cyclic thioesters are highly reactive towards aldehydes in an aqueous environment, forming substituted thiazolidine/thiazinane carboxylic acids. Interestingly, it has been shown that naturally occurring FA undergoes simple, non-enzymatic condensation with Hcy and HTL producing 1,3-thiazinane-4-carboxylic acid (TCA) (see Section 2, Figure 1a) [12,13,14]. Nevertheless, it should be emphasized that very little is known about TCA thus far. Only three reports on TCA are available at present indicating that a relatively stable product, containing the six-membered thiazinane ring from Hcy or its thioester, and FA, is formed in aqueous media over a 5–10 pH range, especially in alkaline solutions representing physiological conditions [12,14]. Additionally, it has been identified that the speed of the reversible equilibrium between Hcy/HTL and FA at a constant temperature is dependent on the pH [14]; while the presence of a mitochondrial preparation [12] and *Aerobacter aerogenes* [13] was found not to affect the rate of the condensation reaction. Interestingly, the problem concerning the presence and determination of Hcy/HTL and FA-derived 1,3-thiazinanes in living systems has received no attention, as yet. Given the fact that such reactions occur in vitro and Hcy/HTL and FA are ubiquitous in human biofluids, one would reasonably expect that TCA would also be present. However, it has not yet been established whether TCA is present in vivo. On the other hand, there is evidence that some thiazolidines are formed in vivo, e.g., cysteine (Cys), a homolog of Hcy, and pyridoxal 5’-phosphate adduct, namely 2-(3-hydroxy-5-phosphonooxymethyl-2-methyl-4-pyridyl)-1,3-thiazolidine-4-carboxylic acid have been shown to be present in human plasma [15]; while 2-methyl-1,3-thiazolidine-4-carboxylic acid, a condensation product of Cys and acetaldehyde, produced as a first intermediate in oxidative ethanol metabolism, has been demonstrated to be present in human plasma and urine as a consequence of alcohol (ethanol) consumption [16].

As a result, the present paper aims to validate findings regarding in vivo formation and presence of TCA in the human body. We selected urine as the matrix of choice, as it is easily accessible and can be obtained in a non-intrusive and non-invasive way; and because it has been demonstrated that in rats injected intraperitoneally with radiolabeled TCA, 55% of the administered dose was excreted in urine and only 6% was expired as carbon dioxide [12]. On the one hand, it may be surprising that our efforts focused on the search for TCA instead of Cys and FA adduct as the concentration of urinary Cys is about 50-times as high as Hcy/HTL in total [17,18]. However, such approach should come as no surprise because W.B. Neely [13] demonstrated that Hcy is much more reactive toward FA than Cys which is reflected in the ease of formation of the less taut six-membered thiazinane ring from Hcy and FA. Important milestones in the validation of the assumption included (1) development of an effective analytical tool based on gas chromatography coupled with a mass spectrometry technique (GC–MS) for the determination of urinary TCA, and (2) the application of the assay to real samples in order to confirm or exclude the performance of the method as well as the presence of TCA in humans. Importantly, the GC–MS technique was assumed to be suitable for this purpose due to its high-throughput potential, sensitivity, specificity, and excellent resolution, along with high degrees of reproducibility and accuracy. This article discusses the essential steps, with some justification, that were taken to achieve the intended objective. Moreover, the advantages, pitfalls, and limitations of the GC–MS assay are mentioned.

## 2. Results and Discussion

Nowadays, civilization diseases are perceived as one of humanity’s most pressing problems. As a result, it has become crucial to identify new biomarkers and put efforts into developing new analytical tools to facilitate large-scale screening of them. In relation to assays based on separation techniques, it is generally known that proper sample handling and management combined with separation and detection conditions play a pivotal role in the quality of generated results. Therefore, detailed experiments were conducted in order to establish that the reported method is reliable for analyte determination purposes. Considerable attention was given to optimizing the procedures and conditions connected with the selective extraction and detection of the analyte. The following sections of the article provide the reader with all necessary information regarding the development, validation, and in-study use of the GC–MS based method for the determination of the newly recognized metabolite of sulfur metabolism, namely TCA, in human urine. Importantly, the role of the HTL/Hcy and FA-derived adduct in the human body still remains substantially unknown. So far, only one piece of evidence can be found in the literature that suggests that facile formation of TCA may diminish toxic Hcy, HTL and FA content in biofluids, providing a beneficial effect on human health. Based on data provided by J. Wriston and C. Mackenzie in the late 1950s [12], it seems reasonable to assume that Hcy, HTL and FA may be disposed of in the form of metabolically inactive TCA, as rats injected intraperitoneally with radiolabeled TCA largely excreted the administered dose in urine.

### 2.1. Sample Preparation

In the presented study, the GC–MS method was designed to determine TCA in human urine. Sample preparation involved chemical derivatization with isobutyl chloroformate (IBCF) in the presence of pyridine, acting as a catalyst, followed by ethyl acetate extraction of the isobutyl derivative of TCA (TCA-IBCF), and immediate analysis. The sequence of events leading up to the preparation of the sample to be analyzed was not accidental since it was assumed that increasing the hydrophobicity of the relatively polar TCA before transferring the molecule into the organic solvent would be valuable for improving extraction efficiency. Firstly, preliminary studies were carried out using the method developed by V. Dufková et al. [19], enabling GC–MS analysis of perfluorinated organic acids in samples of river water. Then, the chemical and flow variables influencing chemical derivatization and the extraction processes were optimized in detail taking account of the different matrix. In general, these experiments were performed using the described procedures herein based on GC–MS measurements (see Section 3.5 and Section 3.6). In each case, the appearance of a product peak on the chromatogram and a comparison of its area was used to determine the particular process efficiency.

#### 2.1.1. Derivatization

As it was envisioned, TCA did not possess the desired physicochemical properties to meet the requirements of the implemented technique. Therefore, chemical modification was a necessary part of the sample preparation process. Interestingly, a wide variety of derivatization methods have been developed to overcome the above-mentioned constraints. Among others, four types of derivatization reactions preceding a GC analysis are most commonly used, namely silylation, acylation, alkylation and esterification methods, to enable analysis of organic compounds with inadequate volatility or thermal stability; as well as to improve their chromatographic behavior or detectability [20,21,22]. In the presented study, considerable attention was paid to the chemical reactions proceeding in media with high water content at room temperature in order to simplify the sample pretreatment procedure and improve batch to batch reproducibility; apart from increasing TCA hydrophobicity before the extraction step, as stated previously. Based on our previous experience with the development of the GC–MS method for urinary HTL determination [23], the use of IBCF was evaluated. It was demonstrated that with the proper use of the above-mentioned derivatization reagent, a successful analysis of a wide range of organic compounds from environmental samples can be achieved [19,24].

Firstly, it was shown that upon derivatization TCA was converted into its corresponding isobutyl derivative, namely TCA-IBCF which produced intense specific fragment ion peaks, suitable for the analyte monitoring (see Section 2.2, Figure 6). In addition, it was found that chemical modification was also valuable for increasing the molecular weight of the analyte which helped separate the GC sample peaks from the solvent front. TCA possessed two potential places accessible to IBCF, provided by the amine functional group and carboxyl group [24]. However, fragmentation ions, found during the study, indicated that the carboxyl group in the molecule remained unmodified under set conditions (see Section 3.5). Importantly, such circumstances did not affect the effective separation and detection of TCA. In relation to TCA, the IBCF-mediated derivatization generally involved the replacement of the active hydrogen on the amine group with the isobutyl formate moiety, followed by hydrochloric acid release under pyridine catalysis, yielding less polar, more volatile and a relatively thermally stable TCA-IBCF derivative. Thus, the most probable schematic of the derivatization reaction of TCA with IBCF is shown in Figure 1b.

**Figure 2:**
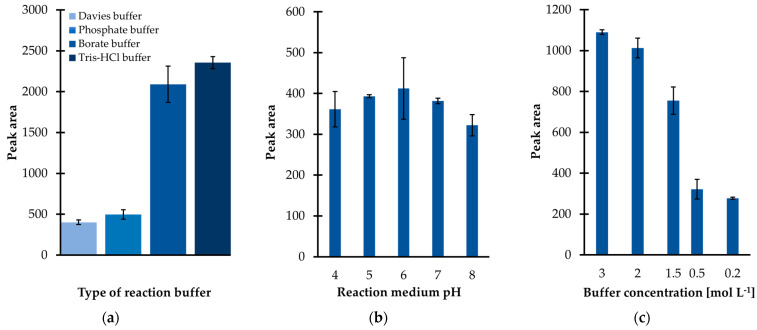
Derivatization reaction yield as a function of (**a**) the type of derivatization buffer; (**b**) pH of the reaction medium; (**c**) tris(hydroxymethyl)aminomethane-hydrochloride buffer (Tris-HCl) pH 7 concentration, expressed as the peak area of the isobutyl derivative of 1,3-thiazinane-4-carboxylic acid (TCA-IBCF). Samples were analyzed according to the procedure described in Section 3.6. Error bars refer to the standard deviation (SD) of the data (*n* = 3). Abbreviations: Tris-HCl, tris(hydroxymethyl)aminomethane-hydrochloride acid buffer.

Since the preliminary research results were promising, subsequently the influence of several factors on derivatization efficiency were carefully studied. Firstly, it was recognized that the yield of the IBCF-mediated acylation was highly dependent on the composition of the reaction medium. Several buffer solutions, including phosphate (PB), borate, tris(hydroxymethyl)aminomethane-hydrochloride (Tris-HCl), and Davies buffer were tested. Among them, the Tris-HCl buffer was found to be superior with regard to derivatization and extraction efficiency since the signal intensity of the TCA-IBCF derivative was greater than that registered when other buffer solutions, at the given pH and total buffer concentration, were used (Figure 2a). As a result, Tris-HCl was chosen to adjust the sample pH regardless of the fact that it contained functional groups, namely hydroxyl and amino groups, that might react with the derivatizing agent of choice [24]. As shown in Figure 5 (see Section 2.2), no interference at the retention time of the analyte originating from Tris-HCl buffer was registered, indicating that the particular buffer was suitable as the derivatization reaction medium.

In parallel, the influence of the pH of the reaction medium on derivatization efficiency was evaluated, as the molecule of TCA can exist in different ionization states, which may have an impact on its reactivity. In addition, it was recognized that the reaction medium pH may not only have an effect on the analyte’s reactivity but also on the efficiency of the subsequent sample preparation step, namely extraction. In the presented study, the effect of pH within the range of 4 to 8 was thus evaluated, corresponding to the expected pH of urine under normal conditions. An universal buffer solution of Davies, which has been demonstrated to be suitable for use over the pH range 2 to 12 [25], was utilized to control the pH level. When analyzing Figure 2b, a moderate pH dependence on process efficiency can be noticed, suggesting that slightly acidic or even neutral conditions are essential to provide the best results. However, a pH lower than 4 or greater than 7, for the solution, may have a negative effect on the derivatization efficiency as the peak area of the TCA-IBCF derivative decreased with decreasing/increasing pH, respectively (Figure 2b). In this way, the limited stability of the obtained derivative in strongly acidic/alkaline aqueous media must be considered. Since the useful pH range of the preselected buffering medium is 7 to 9, Tris-HCl buffer, with a pH of 7 was chosen for further experiments.

Next, experiments were conducted in order to optimize the ionic strength of the buffer solution, which was the next factor affecting the particular processes yield. Different Tris-HCl buffer solutions were prepared at five concentration levels, ranging from 0.2to 3 mol L^−1^. As shown in Figure 2c, a progressive increase in the peak area of the TCA-IBCF derivative was observed in parallel with the rise in concentration of Tris-HCl from 0.2 to 3 mol L^−1^. In addition, the improvement of the precision of the experiment was concomitantly noticed. As a result, a further 3 mol L^−1^ solution of the buffer was used.

Additional experimental work was undertaken to establish the optimal ratio of urine to 3 mol L^−1^ Tris-HCl buffer, pH 7. Taking into account that the pH of the reaction medium affected the chemical derivatization and extraction processes yield, as well as the fact that urine pH can range from 4 to 8 under normal conditions according to the American Association for Clinical Chemistry, an adjustment to the pH of the particular sample was necessary to minimize its contribution to the recovery losses in this method. In order to evaluate the optimal ratio of urine to the buffer solution of choice, a series of three measurements were performed using pooled urine samples, made up of small pools of the specimens from all donations, that were produced in our laboratory for this purpose. Firstly, the pH of the particular sample was adjusted to a specific pH within the pH range of 4 to 8 by adding a few drops of either diluted hydrochloric acid or sodium hydroxide solution to the sample until the desired pH was achieved. Next, experiments were performed to establish the optimal amount of the buffer by mixing urine samples with different volumes of 3 mol L^−1^ Tris-HCl buffer, pH 7. Finally, it was established that a minimum ratio of 1:4, urine to buffer was necessary to achieve a pH for the mixture at the same level as the pH of the buffer added to the sample, regardless of the initial pH of the particular sample or its source. As described in the following sections of the paper, the most satisfactory results were obtained when 50 µL of urine were mixed with 200 µL of 3 mol L^−1^ Tris-HCl buffer, pH 7.

According to the literature [24], alkyl chloroformates are not only influenced by the solvent system, but also by the addition of a catalyst and optional alcohol. In particular, a basic catalyst acting as an acid scavenger is required to neutralize hydrochloric acid, liberated by the derivatization process, in order to drive the reaction forward and/or increase the reactivity of the reagent itself. In parallel, the presence of an auxiliary agent, namely alcohol, in the reaction medium is not prerequisite although it could easily enhance the derivatization yield by means of dispersing the sedimental (cloudy) phase which appears as a result of IBCF and catalyst addition.

In the presented study, one of the most commonly used catalysts, namely pyridine, was employed to facilitate the IBCF assisted reactions. Since pyridine is considered to be toxic, preliminary experiments were conducted to check whether its presence was indispensable for the derivatization. Two sets of samples were prepared; one was treated with IBCF and pyridine, the other with the derivatizing agent itself. Importantly, in the absence of pyridine the outcome of the reaction was rather disappointing. These studies thus have confirmed that the transformation of TCA to the *N*-acylated product with IBCF is promoted by the use of pyridine as an acid-binding agent [24]. Therefore, the influence of the concentration of the catalyst of choice in the study samples on derivatization efficiency was evaluated afterwards. In particular, the effect of the pyridine concentration within the range 0.06 to 0.37 mol L^−1^ was tested, corresponding to the addition of 1.5–9 µL of pyridine per 300 µL of reaction mixture. It was found that the derivatization efficiency was enhanced in parallel with an increase in concentration of the catalyst from 0.06 to 0.25 mol L^−1^ (0.5–2%), and then the peak area of the TCA-IBCF derivative decreased with the further increasing its concentration. Interestingly, the phenomenon of progressive alkyl chloroformate decomposition along with the diminished efficiency of the derivatization process in the presence of excess pyridine seemed to be characteristic for the course of the reaction [24]. As a result, 6 µL of pyridine was found to be optimal and was taken for further experiments. Moreover, it is worth mentioning that higher volumes of the catalyst were excluded as they gave rise to excessive baseline noise.

Subsequently, additional experiments were undertaken to determine whether the presence of alcohol was essential for the derivatization reaction or had a negligible effect on the course of the reaction. Two sets of samples were prepared; the samples were treated with IBCF and pyridine in the presence or absence of the corresponding alkyl alcohol. Importantly, the same process yield was achieved even without alcohol in the reaction medium. For this reason, we decided not to use a dispersant in order to reduce chemical consumption, and instead, we chose to shake the sample to achieve dispersion. Indeed, it was recognized that gently shaking samples by hand was sufficient to effectively modify the analyte. In addition, it needs to be emphasized that when samples were shaken violently, vigorously stirred/vortexed or placed in an ultrasonic bath, it resulted in an explosion and the subsequent loss of the sample.

**Figure 3:**
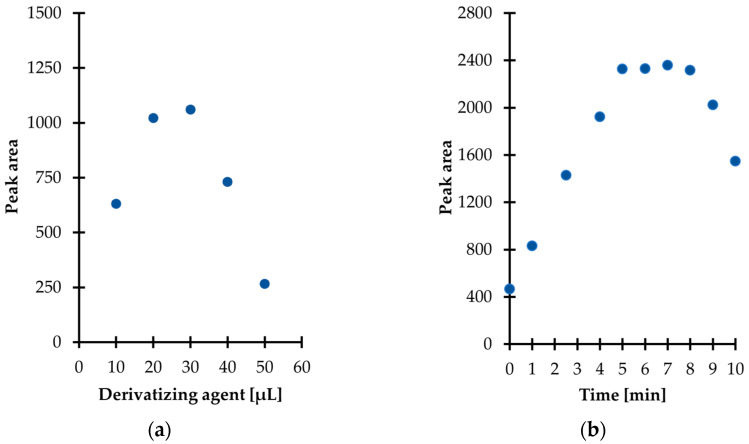
Derivatization reaction yield as a function of (**a**) the amount of the reagent; (**b**) time combined with examination of the isobutyl derivative of 1,3-thiazinane-4-carboxylic acid (TCA-IBCF) stability in the aqueous phase, expressed as a peak area of the derivative. Samples were analyzed according to the procedure described in Section 3.6.

Then, experiments were performed to establish the optimal amount of derivatization reagent for TCA derivatization in urine. Five different quantities, namely 10, 20, 30, 40, and 50 µL were tested, providing the concentration of IBCF in the study samples in the range of 0.27–1.34 mol L^−1^. As shown in Figure 3a, the derivatization efficiency was enhanced in parallel with an increase in the of IBCF from 0.27 to 0.81 mol L^−1^, and then the trend towards higher yield was reversed with further increasing its content in the reaction mixture. Importantly, this phenomenon was not surprising as it has been reported elsewhere that the reaction products may be decomposed when excessive amounts of IBCF over pyridine is used [24]. Therefore, this fact cannot be neglected in any attempt to employ IBCF and other alkyl chloroformates as derivatizing agents as the presence of their excess may result in the occurrence of side reactions along with diminished efficiency of the derivatization process. Finally, 30 µL of IBCF was taken for further experiments as it gave rise to the best results (Figure 3a). Importantly, it has been recognized that pyridine and IBCF have to be added to the reaction mixture separately, since mixing them beforehand results in a cloudy solution which impedes precise pipetting.

Further experiments were conducted in order to establish the derivatization reaction kinetics at room temperature. In parallel, the stability of the obtained derivative in aqueous media was also evaluated. In particular, it was established that the reaction at the above-defined conditions was completed in just 5 min after mixing the reagents (Figure 3b). Therefore, no experimental work was undertaken to further optimize the temperature of the reaction. Notably, it was also found that the TCA-IBCF derivative signal remained stable no longer than 3 min under set conditions, as a progressive decrease of the signal peak area was observed (Figure 3b). Hence, it is highly recommended to subject samples to the subsequent processing step, namely extraction just after completion of the derivatization reaction. It means that the extraction solvent should be added to the reaction mixture within 5 min and no later than 8 min after mixing the reagents together; this was considered well within the sample stability and derivatization efficiency, as well.

In summary, our research led us to the conclusion that Tris-HCl buffer with pyridine proved to be a suitable derivatization reaction medium, while the mere addition of IBCF led to the almost immediate formation of the desirable reaction product. As a result, for routine analysis, 50 µL of urine was initially mixed with 200 µL 3 mol L^−1^ Tris-HCl buffer, pH 7 and was then treated with 6 µL pyridine and 30 µL IBCF, resulting in the final concentration in urine samples of 0.26 mol L^−1^ (~2.1%) and 0.81 mol L^−1^ (~10.5%), respectively. As described above, processing samples for 5 min at room temperature, after gentle shaking by hand, was sufficient to effectively modify the analyte. Importantly, such an approach was also advantageous as it provided good repeatability of the reaction. From a purely practical point of view, an explosion and subsequent loss of the sample was not observed under these conditions. Notably, analyte dilution was not a limitation as samples were concentrated in the subsequent processing step (see Section 2.1.2).

#### 2.1.2. Sample Purification

It has long been known that water-containing samples cannot be directly injected into a GC–MS system without purification prior to analysis. According to the literature, a number of chemical and physical purification methods have been identified, and the most commonly used techniques to adapt sample matrices to be more compatible with the target chromatographic method involve extraction [26].

**Figure 4:**
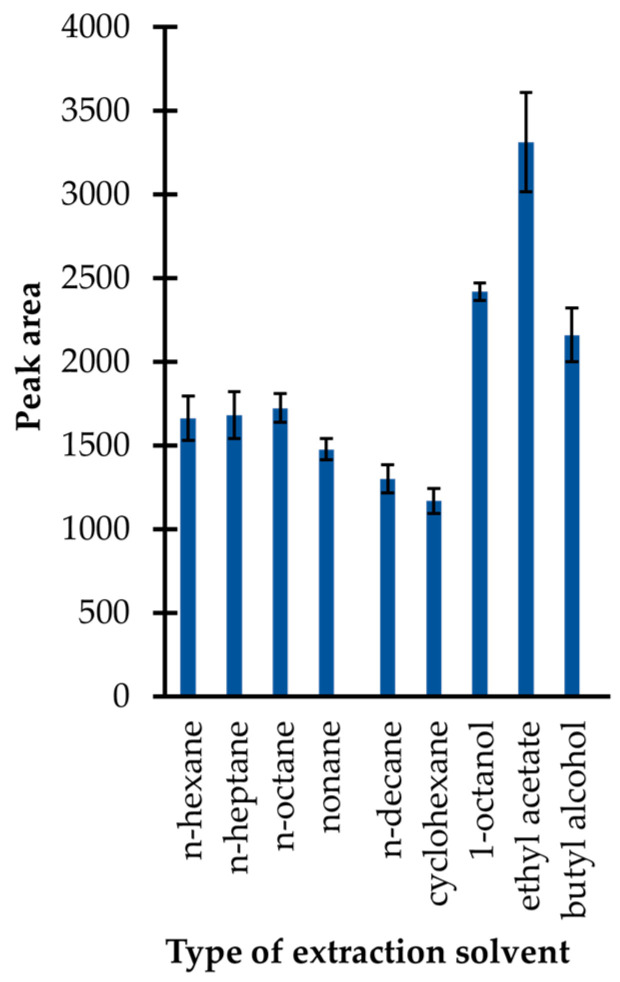
Extraction efficiency of the isobutyl derivative of 1,3-thiazinane-4-carboxylic acid (TCA-IBCF) as a function of the type of extraction solvent expressed as a peak area of the derivative. Samples were analyzed according to the procedure described in Section 3.6. Error bars refer to the standard deviation (SD) of the data (*n* = 3).

In the presented study, an approach utilizing simple liquid–liquid extraction was used. Firstly, several immiscible solvents with lower density than water, including n-hexane, n-heptane, n-octane, nonane, n-decane, cyclohexane, ethyl acetate, 1-octanol, and butyl alcohol were tested. In general, the selection of extractants was made on the basis of findings that the upper organic (acceptor) phase is easier to collect for a GC–MS analysis than the bottom one. As shown in Figure 4, the application of ethyl acetate resulted in the highest extraction efficiency, among other preselected organic solvents. In addition, it was identified that its application resulted in less complex chromatograms in comparison with those registered when other organic solvents were used. Therefore, ethyl acetate was selected.

Then, experiments were performed to establish the optimal amount of the organic solvent of choice. Six different volumes, namely 50, 100, 150, 200, 250 and 300 µL, were tested that amounted to 1 to 6-times the volume of the urine specimen. In particular, it was found that the extraction efficiency decreased with the increase in the extractant volume, due to a substantial dilution of the sample (analyte). Indeed, an efficient extraction was achieved by mixing the resulting mixture with just 50 µL of ethyl acetate, while a minimum volume of 100 µL extractant was found to be necessary to provide both efficient extraction, and sufficiently large enough volume of acceptor phase to enable easy collection for the GC–MS analysis. Thus, 100 µL of ethyl acetate was selected as it was considered satisfactory for ensuring sample handling convenience. 

**Figure 5:**
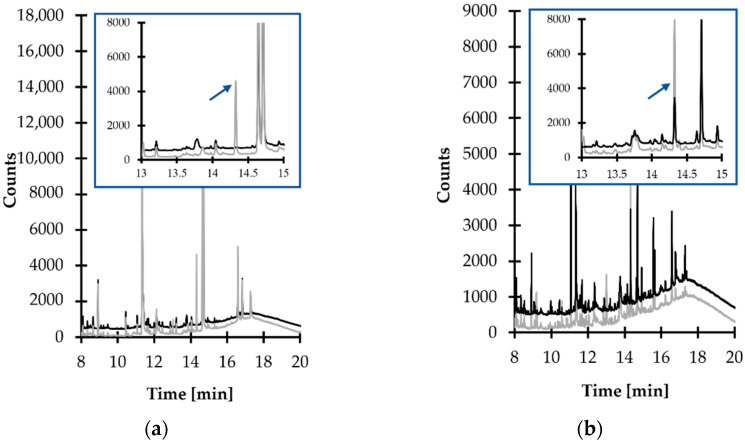
Representative chromatograms of standard solutions and human urine prepared according to the procedure described in Section 3.5. Chromatographic conditions were as described in Section 3.6. (**a**) Blank standard solution (black line) and standard solution of 1,3-thiazinane-4-carboxylic acid (TCA) (50 µmol L^−1^ in urine) (grey line); (**b**) normal human urine sample (black line) and the same sample spiked with the analyte (50 µmol L^−1^ in urine) (grey line). Under these conditions, the peak of the isobutyl derivative of TCA (TCA-IBCF) appears at 14.3 min.

Further experiments were conducted to obtain information about the extraction conditions that promoted the process. In parallel, the stability of the TCA-IBCF derivative in organic media was examined. In particular, it was established that shaking/vortexing the samples followed by centrifugation did not markedly affect operational efficiency, which was beneficial for workflow simplification. Nevertheless, centrifugation was pointed out to be a necessary part of the sample preparation process as signals were higher by about 60% under the discussed conditions in comparison with those registered when the extraction was not accelerated by spinning the samples in solution around an axis at high speed. Notably, it was also found that the extraction under the above-defined conditions was completed in just 1 min after mixing the reagents and immediately placing the samples in the centrifuge. In parallel, it was established that the TCA-IBCF derivative signal remained stable no longer than subsequent 4 min under the set conditions, and then the extraction efficiency decreased dramatically with the prolonged processing time, which was tested in the range of 5–10 min. Equally importantly, the same observations were made when the stability of the study samples in the autosampler, prepared according to the procedure described in Section 3.5, were evaluated. As a result, we were able to conclude from this study that the obtained derivative is fairly unstable under these set conditions, indicating the necessity of subjecting the samples to the GC–MS analysis just after their preparation in order to produce meaningful results. On the other hand, it was important to recognize that the TCA-IBCF derivative did not undergo thermal decomposition in the GC system under optimized conditions (see Section 3.6) and produced a single symmetrical peak (see Section 2.2, Figure 5), although high thermal processing temperatures during GC–MS analysis may potentially lead to the formation of degradation products [27]. Finally, for routine analysis, 286 µL of the resulting mixture and 100 µL of ethyl acetate were mixed together, and then placed in the centrifuge at 6000 rpm for 1 min at room temperature. Under these conditions, the cloudy (upper) organic phase was obtained, which was then collected and injected into the GC–MS system immediately.

In summary, these experiments established an optimal procedure, in which urine was treated with a derivatizing agent, namely IBCF in the presence of pyridine, and subjected to the GC–MS system without delay after ethyl acetate extraction. Importantly, this is a simple one-pot sample processing method for urinary TCA determination, in which the overall sample preparation time was estimated to be 10 min, taking into account all the operations that need to be performed, including derivatization, extraction, centrifugation, and pipetting etc. Furthermore, the process was accompanied by consumption of 336 µL of inexpensive chemicals. In our opinion, the GC–MS assay can thus be considered environmentally-friendly thanks to the possibility of carrying out the chemical analysis on a very small scale combined with low consumption of hazardous chemicals and laboratory disposable plasticware. Nevertheless, it needs to be emphasized that a successful analysis using the proposed method can only be achieved when the recommended sample handling and management procedures, described herein, are followed.

### 2.2. GC Separation and MS Detection

Careful optimization of separation and detection conditions were performed in the next stage of the method development process, while the initial experiments were conducted with the use of a GC–MS method dedicated to urinary HTL measurements [23]. As it was recognized that the target compound peak was not well-resolved under these conditions, a standard approach was employed to specify optimal conditions by assessing the influence of many operating parameters of the GC–MS system on the method’s performance. During the study, crucial rules have been pointed out to be followed in order to perform a successful analysis. Particularly, it was recognized that an initial temperature no higher than 100 °C, as well as temperature programmable conditions accompanied by increasing temperature in a maximum ramp rate estimated as 15 °C per hold-up time, were essential to maintain the efficient resolution of the TCA-IBCF derivative peak from other sample components. In addition, it was found that a ramp to 300 °C followed by slow cooling down was necessary to properly equilibrate the GC–MS system between analyses. This standard procedure, which went along with five sample washes and five syringe primes prior to the injection, was also essential to reduce carryover to the minimum level. Under optimized conditions (see Section 3.6), providing the baseline separation, the peak of the TCA-IBCF derivative eluted within 14.3 min and was easy to distinguish from the responses of all the concomitant matrix components. Equally importantly, each time the elution profile of blank samples was free from any interference at the retention time of the analyte (Figure 5).

**Figure 6:**
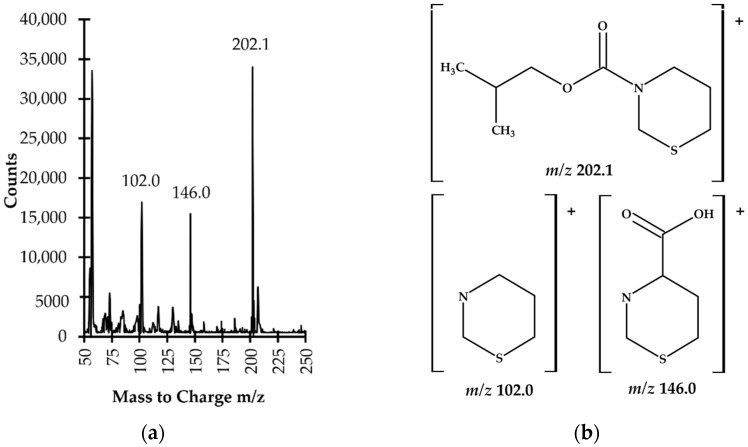
(**a**) Electron ionization (EI) mass spectrum of 1,3-thiazinane-4-carboxylic acid (TCA) isobutyl derivative (TCA-IBCF) obtained by analyzing a standard solution of TCA (60 µmol L^−1^) prepared according to the procedure described in Section 3.5; (**b**) Proposed fragment ion structures in the EI mass spectrum of TCA-IBCF derivative.

The identification and confirmation of the target compound were performed by analyzing the standard solution of TCA (60 µmol L^−1^) processed according to the procedure described in Section 3.5. The analyses were carried out by the GC–MS system which was initially set to acquire data in scan mode and subsequently in selected ion monitoring mode (SIM). The electron ionization (EI) mass spectra were recorded by setting the instrument to gather data, stepping the mass filter within *m*/*z* 50–450 range. Three ions, namely *m*/*z* 102.0, *m*/*z* 146.0 and *m*/*z* 202.1 were preselected as suitable for analyte monitoring (Figure 6).

In order to increase sensitivity and selectivity in the trace analysis, further analyses were conducted with SIM MS mode, using *m*/*z* 102.0 and *m*/*z* 146.0 to identify the target compound as well as *m*/*z* 202.1 as a quantification ion. The confirmation of the origin of the 14.3 min peak and quantification of the compound of interest in real samples were based upon comparison of retention time and specific ions with a corresponding set of data obtained by analyzing an authentic compound. Finally, satisfactory method selectivity was achieved by means of selecting the oven temperature program and the specific ions monitored by a MS detector. As shown in Figure 5a,b, representing SIM-chromatograms registered under optimized GC–MS conditions (see Section 3.6), the TCA-IBCF derivative produced a single symmetrical peak which was well-resolved from other peaks on the capillary column coated with a HP-5MS phase. Equally importantly, potentially existing thermal effects at elevated temperatures on the study samples’ components within the GC system, which is a well-known but accepted problem [27], did not detrimentally affect the accuracy, precision or sensitivity of the method, as it was demonstrated during the validation and implementation of the proposed GC–MS assay (see Section 2.3). Therefore, no experimental work was undertaken to evaluate the thermal effects of particular heated GC–MS system elements on the stability of the TCA-IBCF derivative.

### 2.3. Validation of the Method

In order to prove that the optimized method was suited to the analysis of the study samples, full validation of the new method for the analysis of urinary TCA was conducted. In general, the same approach was used for this purpose as was reported in our earlier papers [15,28]. The elements and acceptance criteria of the method development and validation were selected based on the United States Food and Drug Administration guidance for bioanalytical methods validation [29]. In particular, essential parameters, measured in combined experiments, such as selectivity, linearity, the limit of quantification (LOQ), accuracy, and precision were evaluated. In parallel, the matrix effect was investigated during the validation and implementation of the GC–MS assay. In addition, system suitability was assessed.

#### 2.3.1. System Suitability

System suitability parameters such as repeatability of retention time expressed as the coefficient of variation (CV) of retention time, asymmetry factor, and number of theoretical plates were selected to determine instrument performance under optimized conditions (see Section 3.6). System suitability test calculations were performed by analyzing a standard solution of TCA (50 µmol L^−1^ in urine) in 10 replicate injections. Importantly, successful system suitability test runs indicated that the system was performing in a manner that led to the production of accurate and reproducible data. Detailed data regarding the system suitability tests are summarized in Table 1.

#### 2.3.2. Selectivity

Some attempts have been made to verify the selectivity of the analyte in the presence of concomitant matrix constituents. In particular, selectivity studies assessed interferences originating from matrix components, such as Hcy, HTL and FA, being precursors of TCA formation, which have been recognized to also be present in urine specimens [7,17,18]. Firstly, a blank standard solution and a standard solution of TCA (50 µmol L^−1^ in urine) were prepared and analyzed according to the procedure described in Section 3.5 and Section 3.6, respectively. As shown in Figure 5a, the elution profile was free from any interference at the retention time of the analyte. Equally importantly, the same observations were made when the standard solution of Hcy, HTL and FA (50 µmol L^−1^ in urine) were assayed. Under these conditions, the peak of Hcy/HTL-IBCF derivative appeared at 9.3 min, while the appearance of a product peak of the FA-IBCF derivative on the chromatogram was not noted. Afterwards, normal urine samples from six individual sources and the same samples spiked with Hcy, HTL and FA (50 µmol L^−1^ in urine) were assayed according to the procedures described herein (see Section 3.5 and Section 3.6). In all cases, no increase in the peak area of the target compound was observed. In addition, the analyte peak was evaluated for purity. For this purpose, the MS detector was set to acquire spectra online throughout the entire chromatogram, and the spectra obtained during the elution of the target compound peak were compared. Importantly, the same spectra, acquired in different sections of the TCA-IBCF peak were observed, indicating its purity.

#### 2.3.3. Linearity

A typical approach, namely the external standard calibration method, was used for the calibration of the method. For this purpose, multilevel calibration curves were generated and run in triplicate over five subsequent working days. The calibration curves consisted of a blank sample and six calibration standards, covering the expected unknown TCA concentration in study samples, in the range of 1–50 µmol L^−1^ in urine, including LOQ. Calibrators were prepared in laboratory-made pooled urine by spiking the matrix with known quantities of the target compound. Pooled urine made up of small pools of the specimens from all donations was produced in our laboratory. Since urine samples free of TCA were not available, the endogenous concentration of the analyte was evaluated before the calibration curve preparation by triplicate analysis. All concentrations were tested with the use of calibration curves prepared daily, especially on that occasion. The linearity was initially evaluated graphically by visually inspecting a plot of the peak area as a function of TCA concentration. Importantly, it was established that only the signal peak area of the TCA-IBCF derivative increased linearly with the growing concentration of TCA. Afterwards, a mathematical evaluation was conducted using the least-squares regression model to describe the concentration–response relationship. In particular, curves’ correlation coefficient (R) was monitored showing that the instrument response was directly proportional to the TCA concentration within the intended quantitation range. Moreover, substantial changes in the slope of the particular regression line across a day were not observed (Table 2). Nevertheless, it was discovered that the analytical method could be affected by matrix components. Therefore, matrix effect was investigated during the validation and implementation of the method (see Section 2.3.4).

#### 2.3.4. Matrix Effect

The matrix effect evaluation involved comparing calibration curves of the multiple sources of urine samples against a calibration curve of the pooled matrix. Importantly, it was recognized that the calibration curves created from the pooled matrix markedly differed from the ones prepared in human urine samples from the six individual sources. In particular, the slope of the regression lines deviated by more than 12.5%, indicating the presence of a matrix effect. Due to this variation, a (single) standard addition method was used to establish the levels of TCA in the urine samples, instead of using the traditional calibration curve approach, in order to improve the procedure’s reliability.

#### 2.3.5. Accuracy and Precision

Accuracy and precision of the assay were evaluated under the described conditions in order to assess the variability associated with the measurements. The precision was expressed in the form of CV, whereas the accuracy as the percentage of analyte recovery calculated by expressing the mean measured amount as a percentage of the added amount using the following formula:

Accuracy (%) = [(measured amount − endogenous content)/added amount] × 100,(1)

The evaluation of the above-mentioned parameters was carried out at two levels, and experiments were completed as a part of a linearity assessment. Intra-assay precision and accuracy were demonstrated by triplicate analysis of freshly prepared calibrators. They referred to the pooled urine samples enriched with TCA at three different levels, covering quantitation range, including one close to the lower LOQ, one in the middle of the range, and one at the upper LOQ. Experiments for estimating intermediate accuracy and precision were repeated, in the same manner, over five subsequent days. All concentrations were tested with the use of calibration curves prepared especially on that occasion. Importantly, results obtained from the analytical runs met the acceptance criteria. In particular, the accuracy ranged from 92.05% to 104.56% and 86.48% to 104.35% for intra- and inter-day variation, respectively. The precision did not exceed 13.01% of CV at any examined concentration level and varied from 3.03% to 12.97% and 1.59% to 13.01% for intra- and inter-day measurements, respectively. Notably, such good precision and accuracy were obtained with no outliers excluded. Detailed data on precision and accuracy from the five-day experiments, compared with intra-assay precision and accuracy, are shown in Table 3.

#### 2.3.6. The Limit of Quantification

In the presented study, the LOQ evaluation was done as a part of the intra-assay precision and accuracy assessment for the calibration range. In particular, the lowest concentration on the calibration curve, which equals 1 µmol L^−1^, was recognized as the LOQ. Indeed, this concentration of the analyte produced a detector response which was clearly distinguished from the baseline and reproducible with a precision that did not exceed 12.97%, and accuracy ranged from 92.05% to 104.56%. Interestingly, the estimated LOQ value corresponded to the LOQ determined experimentally by the signal-to-noise method. In this method, a proxy matrix (0.9% sodium chloride in 0.1 mol L^−1^ PB pH 7.4) was enriched with decreasing concentrations of the analyte and treated according to the procedure described in Section 3.5 and Section 3.6 until the injected amount of TCA resulted in a peak 10-times as high as the baseline noise level. Importantly, this value is the first ever reported.

In the end, the method validation proved the suitability of the optimized GC–MS assay for the analysis of the study samples. Particularly, it was demonstrated that the delivered analytical method was sensitive enough and had suitable levels of precision, accuracy and linearity, falling within acceptable tolerance limits [29]. Nevertheless, an excessive impact of the matrix on the assay results was encountered throughout the validation of the method, indicating the need to use a single standard addition method to establish levels of TCA in urine samples. Detailed data regarding all validation parameters, that were evaluated as a part of the linearity assessment using an external standard calibration method, are shown in Table 2 and Table 3.

### 2.4. Application of the Method

In order to prove the utility of the method as well as to confirm or exclude the presence of TCA in humans, the validated GC–MS assay was applied to urine samples donated by volunteers. Fifteen adult individuals were involved in the experiment (eight women and seven men in the 23–73 years age group, providing an average age for the experimental group of 40.53 years). Biological samples taken for the purpose of the research project were handled according to the procedures described in Section 3.4, Section 3.5 and Section 3.6. Importantly, the analyte was detected in all study samples. Thus, the method was also used for quantitative determination of TCA in the collected urine samples. A single standard addition method was used to establish the urinary levels of TCA due to the matrix effect, and the endogenous concentrations of the analyte were evaluated based on data obtained by triplicate analysis of a particular blank urine sample from an individual source and the same sample was spiked with a known quantity of the analyte, providing the concentration of TCA in the study sample of 10 µmol L^−1^ [30]. The concentration of the analyte in each sample was calculated using the following formula:

C_x_ = [Y_x_ × C_s_/(Y_s_ − Y_x_)], (2)

where C_x_—concentration of the analyte in urine sample; Y_x_—analytical signal for the sample containing only the analyte; C_s_—concentration for the sample with the addition of a known amount of the standard; Y_s_—analytical signal for the sample with the addition of a known amount of the standard.

The estimated concentrations of urinary TCA varied from 0.18 to 6.92 µmol L^−1^ with average values of 2.17 ± 2.04 µmol L^−1^ for all donors. Importantly, these values for urinary TCA are the first ever reported. To the best of our knowledge, no reference method thus far has been available to estimate TCA content in human biofluids, in particular urine. The presented GC–MS assay is the first one dealing with the above-mentioned issue. The detailed data on the experimental group and results of the TCA concentration in all assayed samples are shown in Table 4.

## 3. Materials and Methods

### 3.1. Reagents and Materials

All chemicals used throughout this study were commercially available and of analytical reagent grade. TCA, Tris, sodium hydrogen phosphate dihydrate, sodium dihydrogen phosphate dihydrate, sodium tetraborate, citric acid monohydrate, potassium dihydrogen phosphate, potassium chloride, IBCF, anhydrous pyridine, sodium chloride, nonane, n-octane, organic solvents suitable for high performance liquid chromatography (HPLC), namely methanol (MeOH), n-hexane, n-heptane, cyclohexane, ethyl acetate, and 1-octanol were from Sigma-Aldrich, (St. Louis, MO, USA). Hydrochloric acid, boric acid, and sodium hydroxide were from J.T. Baker (Deventer, The Netherlands), while organic solvents suitable for GC, namely, n-decane and butyl alcohol were purchased from Honeywell Fluka (Darmstadt, Germany). Deionized water was produced in our laboratory.

### 3.2. Instrumentation

An Agilent 7820A GC system equipped with automated sample injector model 7693A and MS detector 5977B (Agilent Technologies, Waldbronn, Germany) was used for the GC experiments. The GC apparatus was equipped with a split/splitless inlet, working in a split ratio of 10:1 mode to a 60 m × 0.25 mm HP-5MS quartz capillary column with a 0.25 µm film thickness (Agilent Technologies, Waldbronn, Germany). Data acquisition and analysis were performed using a MassHunter 5977B MSD Bundle with 7820 GC software NIST MS Spectral Library version 2.3.

For sample shaking, Multi-Speed Vortex MSV-3500 (Biosan, Riga, Latvia) was used. During the study, a Mikro 220R centrifuge with fast cool function (Hettich Zentrifugen, Tuttlingen, Germany), and a FiveEasy F-20 pH-meter (Mettler Toledo, Greifensee, Switzerland) were also used. Samples were stored in an ultra-low-temperature freezer (Panasonic Healthcare Co., Ltd., Sakata, Japan). Water was purified using a Milli-QRG system (Millipore, Vienna, Austria).

### 3.3. Stock Solution of TCA

The stock solution of TCA (0.1 mol L^−1^) was prepared daily by dissolving an appropriate amount of TCA powder in HPLC-gradient grade MeOH. Then, the solution was kept at 4 °C for no longer than 24 h. The working solutions of TCA were prepared freshly by dilution of a standard solution with HPLC-gradient grade MeOH as needed and were processed without delay.

### 3.4. Urine Samples Collection

First, early morning urine samples (about 5 mL) were collected from individuals after overnight fasting using a standard method [31]. Samples of urine “mid-stream” were obtained by asking donors to put fluid into a sterile container. Then, samples were cooled on ice and delivered to the laboratory. Importantly, obtained samples were processed without delay using the procedure described in Section 3.5.

A group of fifteen apparently healthy individuals, belonging to an ethnically homogeneous group, was studied. Donors were not supplemented with the analyte, neither Hcy, HTL and/or FA before sample collection. In addition, no medications were allowed. The study was approved by the Ethics Committee of the University of Lodz (decision identification code 4/KBBN-UŁ/III/2020-21, date of approval 27 April 2021). All subjects gave their written informed consent.

### 3.5. Urine Samples Preparation for TCA Quantification by GC–MS

The urine sample (50 µL) was mixed with 200 µL of 3 mol L^−1^ Tris-HCl buffer, pH 7. Then, 6 µL of pyridine, serving as a catalyst, and 30 µL of IBCF were added to the resulting mixture. Thereafter, the mixture was shaken by hand and incubated at room temperature for 5 min. In the next step, the TCA-IBCF derivative was extracted from the sample with 100 µL of ethyl acetate by keeping it in a centrifuge at 6000 rpm for 1 min at room temperature. After centrifugation, the cloudy organic phase (upper layer) was transferred to a vial, and 1 µL of the sample was injected into the GC–MS system without delay. Each sample was analyzed according to the procedure described in Section 3.6. Cautions: Vigorous stirring/vortexing is not recommended to avoid an explosion.

### 3.6. GC–MS Conditions

Helium (99.9999%) was used as the carrier gas with a constant flow rate of 1 mL min^−1^. The injection port temperature was set to 270 °C. The chromatographic separation of the TCA-IBCF derivative was accomplished under thermal gradient conditions. The initial oven temperature was set to 100 °C and increased to 300 °C in steps of 15 °C min-^1^, then held at 300 °C for 3 min. Afterwards, the oven was slowly cooled down in steps of 55 °C min^−1^. The MS detector was operated in the EI mode at 70 eV. The ion source temperature was set at 230 °C, the temperature of quadrupole was set at 150 °C, while the MS interface was set to 250 °C. The multiple ion detector was focused on ions that represented only the portion of the TCA-IBCF derivative with masses of *m*/*z* 102.0, *m*/*z* 146.0 and *m*/*z* 202.1 to identify the target compound, while *m*/*z* 202.1 was preselected as a quantification ion. The instrument was set to acquire data within a 8–20 min range of time, with a dwell time that yielded 15 to 20 scans across the chromatographic peak. Cautions: The derivatizing agent is toxic and corrosive, thus five syringe washes with 3 µL of ethyl acetate prior and after each injection, are highly recommended for corrosion protection for the metal part of the syringe.

## 4. Conclusions

Based on a thorough review of existing publications, it can be reasonably stated that this report generates brand new information on a novel product of sulfur metabolism in humans, namely TCA. In particular, the experiments produced credible evidence that HTL/Hcy and FA adduct was present in human urine. Furthermore, the analytical knowledge of 1,3-thiazinane derivatives was extended. A new method for the assessment of TCA content in human biofluids, namely urine has notably been provided. In parallel, valuable information on the essential stages of the analytical process during 1,3-thiazinane derivative determination has been delivered. In addition, some preliminary conclusions concerning TCA content in apparently healthy donors regardless of sex, age, general state of health etc., were reached. Now, it remains to be investigated and to find an answer as to whether TCA plays a key role in living systems and what kind of role it might be. In fact, the proposed GC–MS approach is not free from restrictions (see Section 2) and the rules described herein should be followed in order to produce meaningful results. In particular, it is highly recommended to quantify the samples using the single standard addition method, which limits the number of samples analyzed per day and assay them without delay due to the limited stability of the TCA-IBCF derivative under experimental conditions. Nonetheless, we hope that the elaborated method will be suitable for meeting this objective as these results are undoubtedly desirable from the standpoint of human well-being. Hopefully, the obtained data will feature in further research in the field, in particular involving biostatistical analysis of medical data in near future.

## Figures and Tables

**Table 1:** System suitability test (*n* = 10).

Parameter	Acceptance Criteria	Value
CV of retention time	≤1%	0.018%
Asymmetry factor	0.8–1.5	1.11
Number of theoretical plates	≥2000	212,846

Abbreviations: CV, coefficient of variation.

**Table 2:** Validation data corresponding to intra-assay measurements (*n* = 3).

Regression Equation	R	CV Slope (%)	Linear Range (µmol L^−1^)	Intra-Assay Precision (%)		Intra-Assay Accuracy (%)		LOQ (µmol L^−1^)
				Min	Max	Min	Max	
y = 37.13x + 17.35	0.9994	3.43	1–50	3.03	12.97	92.05	104.56	1

Abbreviations: CV, coefficient of variation; LOQ, limit of quantification; R, correlation coefficient.

**Table 3:** Precision and accuracy data (*n* = 5).

Concentration (µmol L^−1^)	Precision (%)		Accuracy (%)	
	Intra-Assay	Intermediate	Intra-Assay	Intermediate
1	4.80	3.69	92.05	88.10
20	3.03	13.01	97.66	95.32
50	12.55	7.07	99.06	98.33

**Table 4:** Concentration of 1,3-thiazinane-4-carboxylic acid (TCA) in human urine (*n* = 15).

Volunteer			TCA Concentration ± SD (µmol L^−1^)
No.	Gender	Age (Year)	
1	Female	23	0.49 ± 0.04 ^a^
2	Female	32	1.76 ± 0.05
3	Male	30	0.86 ± 0.01 ^a^
4	Male	36	2.40 ± 0.02
5	Female	51	0.80 ± 0.10 ^a^
6	Female	50	2.28 ± 0.02
7	Female	23	0.61 ± 0.02 ^a^
8	Male	23	0.34 ± 0.05 ^a^
9	Female	23	3.37 ± 0.46
10	Male	50	0.58 ± 0.07 ^a^
11	Female	49	0.18 ± 0.03 ^a^
12	Male	50	2.11 ± 0.12
13	Female	68	5.27 ± 0.07
14	Male	73	4.51 ± 0.14
15	Male	27	6.92 ± 0.72

^a^ Concentration below limit of quantification (LOQ). Abbreviations: TCA, 1,3-thiazinane-4-carboxylic acid; SD, standard deviation of the data (*n* = 3).

## Data Availability

Data is contained within the article. In addition, any obtained data which contributed to the article can be made available by the corresponding author (J.P.) upon request as long as the request does not compromise intellectual property interests.

## Abbreviations

AD	Alzheimer’s disease
Cys	cysteine
CV	coefficient of variation
CVD	cardiovascular disease
DM	diabetes mellitus
EI	electron ionization
FA	formaldehyde
GC	gas chromatography
Hcy	homocysteine
HPLC	high performance liquid chromatography
HTL	homocysteine thiolactone
IBCF	isobutyl chloroformate
LOQ	limit of quantification
MeOH	methanol
MS	mass spectrometry
PB	phosphate buffer
R	correlation coefficient
RT	room temperature
SD	standard deviation
SIM	selected ion monitoring mode
TCA	1,3-thiazinane-4-carboxylic acid
TCA-IBCF	isobutyl derivative of 1,3-thiazinane-4-carboxylic acid
Tris-HCl	tris(hydroxymethyl)aminomethane-hydrochloride acid buffer
